# Ezrin–Radixin–Moesin Binding Phosphoprotein 50: A Potential Novel Biomarker in Human Papilloma Virus-Associated Head and Neck Squamous Cell Carcinomas

**DOI:** 10.1007/s12105-018-0937-z

**Published:** 2018-05-30

**Authors:** Athiva Shankar, Dorothy H. Crouch, Michaelina Macluskey

**Affiliations:** Unit of Oral Surgery and Medicine, University of Dundee Dental School, Park Place, Dundee, DD1 4HN UK

**Keywords:** EBP50, Head and neck squamous cell carcinoma, Human papilloma virus, Oropharyngeal cancers

## Abstract

High-risk human papilloma virus (HR-HPV) has increasingly been associated with head and neck squamous cell carcinoma (HNSCC), in particular oropharyngeal cancers. Ezrin–Radixin–Moesin Binding Phosphoprotein 50 (EBP50), a putative tumour suppressor, localises to the plasma membrane in suprabasal epithelium and to the cytoplasm in proliferative basal layers, and is a target for degradation by the HR-HPV E6 oncoprotein. The aim of this study was to investigate EBP50 protein expression patterns in HNSCC in a large Scottish cohort to determine if there was a correlation with HPV status and clinical outcomes. EBP50 expression patterns were assessed in 156 HNSCC including oropharyngeal (37.8%), laryngeal (24%), oral (19%) and other sites (18.5%), which were genotyped for presence of HR-HPV. HNSCC were generally negative for membranous EBP50. EBP50 expression was either cytoplasmic/absent, being ‘predominantly cytoplasmic’ in 76 (49%), ‘weak/negligible cytoplasmic’ in 44 (28%), ‘strongly cytoplasmic’ in 5 (3%), ‘heterogeneous’ in 26 (17%) and ‘other’ in 5 (3%) samples. Forty tumours (25%) were positive for HPV DNA, predominantly HR-HPV 16, and 44 (28%) were p16 positive. The majority of tumours (71%) with ‘weak/negligible cytoplasmic’ EBP50 expression originated in the oropharynx were more likely to have positive neck nodes, overexpression of p16 and positive tumour HR-HPV status (*P* < 0.001). Differences in EBP50 levels between oropharyngeal and non-oropharyngeal tumours may be linked to degradation of EBP50 by HR-HPV, and loss of EBP50 may therefore be a surrogate biomarker for HR-HPV infection in oropharyngeal tumours.

## Introduction

In 2014, there were 11,400 new cases of head and neck squamous cell carcinoma (HNSCC) in the United Kingdom and over the last decade, incidence rates have increased by almost a quarter [1]. Despite improvements in treatment, almost half the affected patients will not survive 5 years and mortality rates are set to rise to seven deaths per 100,000 people by 2035 [1]. While smoking and alcohol are the biggest risk factors, high-risk human papilloma virus (HR-HPV) is implicated in the increasing rates of oropharyngeal cancer [2].

Ezrin–Radixin–Moesin (ERM) Binding Phosphoprotein 50 (EBP50), a PDZ (postsynaptic density 95, PSD-85; discs large, Dlg; zonula occludens-1, ZO-1) domain scaffolding protein, also known as Na^+^/H^+^ exchanger 3 regulatory factor 1 (NHERF1), is found in abundance at the plasma membranes of polarised epithelial cells where it regulates tissue architecture and cell migration. It functions as a molecular scaffold, promoting the assembly of macromolecular signalling protein complexes at the plasma membrane of epithelial cells, thereby regulating their activity. Major signalling pathways regulated by EBP50 include Phosphatidylinositol-3-OH kinase (PI-3K)/AKT/Phosphatase and tensin homologue (PTEN) pathway, platelet derived growth factor receptor (PDGFR), epidermal growth factor receptor (EGFR) and Wnt/β-catenin signalling [3].

Increasingly, while EBP50 is being reported as a potential player in cancer, its precise role is controversial [3]. Under physiological conditions, EBP50 localises to the apical membrane of polarised epithelial cells where it stabilises transmembrane receptors and junctional complexes [3]. In contrast, in numerous malignant tumours including breast, colorectal and hepatocellular carcinoma, aberrant EBP50 expression (either overexpressed, altered subcellular localisation or loss) has been observed [4–6]. These expression patterns led to the speculation that the function of EBP50 may be dependent on its subcellular location, whereby it acts as a tumour suppressor at the plasma membrane or acts as an oncogene when relocalised to the cytoplasm or its expression is lost [3]. Indeed, there is evidence for both, with cytoplasmic or membranous EBP50 reported to interfere with specific signalling pathways that either promote or suppress tumourigenesis respectively [3–5, 7, 8].

EBP50 is a known target for degradation by the HR-HPV type 16 E6 oncoprotein [9], which contributes to carcinogenesis by disrupting multiple cellular pathways [10]. HPV16 is linked to cervical cancer development [11], and EBP50 has been shown to be downregulated in HPV16-positive cervical premalignant lesions and in cervical cancer-derived cell lines, concomitant with EGFR activation [12].

Oropharyngeal squamous cell carcinoma (OPSCC) is among the cancers with the fastest increasing incidence in Scotland [13] and the United States [14]. A recent United Kingdom study reported that 51.8% of these cancers were HPV-positive [15]. At other head and neck sites, however, HPV is not thought to be causative [16].

The aim of this study was to investigate EBP50 protein expression patterns protein in HNSCC in a large Scottish cohort, to determine if there was a correlation with HPV DNA status and clinical outcomes.

## Materials and Methods

This retrospective study, approved by the Tayside Tissue Bank (TR000325), was conducted according to the guidelines outlined by the Research Governance Framework in Tayside, Scotland. Patients with HNSCC treated between January 2006 and December 2011 were selected from a single centre secondary care hospital. The inclusion criteria were patients over the age of 18 years diagnosed with a new primary HNSCC with adequate tissue available to allow immunohistochemistry (p16, EBP50) and PCR (HPV). The exclusion criteria included patients under 18 years of age, recurrent HNSCC, cases lost to follow up and inadequate quantity/quality of tissue for downstream processing.

The most widely used assay to detect the presence of transcriptionally active HPV in HNSCC is to determine the p16 status of tissue specimens by immunohistochemistry [17]. At this time all oropharyngeal cases had p16 staining carried out as part of the routine pathology reporting of cases in Tayside. A request was made for the tumours for the other head and neck sites to also have p16 staining. Slides stained for p16 were analysed by a single pathologist and scored as p16 positive if strong diffuse nuclear and cytoplasmic staining was observed in ≥ 70% of the tumour, consistent with the reporting protocol within Tayside [18]. Normal tonsillar tissue was used as a control.

Survival data and clinical information were recorded by reviewing case notes. Survival outcomes were assessed against p16 status, smoking and alcohol history, disease stage, HPV status and EBP50 expression. A completed database was transferred to Tayside Health Informatics Centre (HIC) for anonymisation via a secure NHS transfer system.

### Immunohistochemistry

Coupes (5 μm) of formalin fixed paraffin embedded (FFPE) archival tissue were de-paraffinised in xylene, and re-hydrated in graded ethanol solutions (100, 90, 70%). The sections were rinsed with dH_2_O before microwave antigen retrieval in 10 mM citrate buffer (pH 6.4) for 15 min. Endogenous peroxidase activity was blocked with 10% hydrogen peroxide. After rinsing, staining was performed using the Vectastain Elite ABC kit according to the manufacturer (Vector Labs). Briefly, the slides were blocked with normal goat serum [20 min, 1% BSA/PBS (w/v)], before incubating with primary EBP50 rabbit antibody (PA1-090, Thermo Fisher Scientific, 1:2000, 1% BSA/PBS (w/v), 30 min). After washing and incubating with biotinylated secondary antibody (1:200, 1% BSA/PBS (w/v), 30 min) the slides were incubated with Vectastain ABC reagent. After rinsing, the sections were developed in peroxidase substrate solution (5–8 min), rinsed in water, counterstained with haematoxylin, dehydrated in graded ethanol solutions (70, 90, 100%) and cleared in xylene. Tissue sections were mounted with DePeX mounting medium before visualising under a light microscope.

### Quantification of EBP50 Staining

The protocol for scoring of EBP50 immunohistochemistry was adapted from Lv et al. [19]. Sections were analysed by two independent observers (AS and DHC) blinded to the sample identities. Additionally, 10% of the sections were scored by a third independent observer (MM). Differences in inter-observer scores (3 out of 156 tumours, < 2%) were reconciled by re-reviewing sections. Five random representative fields (100 cells each) were viewed using ×40 magnification. Membrane and cytoplasmic EBP50 expression patterns were scored separately as shown in Table 1.

**Table 1:** Quantification of immunohistochemical EBP50 staining Reproduced with permission from Lv et al. [19]

Membrane staining	
Negative	No staining in > 10% of the tumour cells
Positive	Weak or moderate staining in > 10% of the tumour cells
Mixed	Combination of the above
Cytoplasmic staining	
Negligible/weak	Negligible/weak staining in > 10% of the tumour cells
Positive	Moderate staining in > 10% of the tumour cells
Mixed	Combinations of the above

Five random representative fields of 100 cells each were viewed using ×40 magnification, and each tumour allocated a staining pattern for EBP50 after quantification by two independent observers (AS, DHC). A third independent observer (MM) scored 10% of the samples

### DNA Extraction

Tissue section curls (3–5) (5–10 µm) were used for preparation of genomic DNA using the QIAamp DNA FFPE Tissue kit according to the manufacturer’s instructions. Briefly, paraffin was removed with xylene and ethanol. After resuspension in Buffer ATL, the cell pellet was treated with Proteinase K (56 °C, 1 h, then 90 °C, 1 h). After ethanol precipitation, DNA was collected on a QIAamp MinElute column before elution in Buffer ATE. Purified DNA samples were stored at − 20 °C.

### HPV Genotyping of Tumour Samples

HPV DNA was detected by conventional PCR. Consensus primers specific to the β-globin gene and L1, E1 and E7 genes of the HPV virus were used. All β-globin-positive samples were initially subject to PCR with L1 primers. As the L1 open reading frame may be disrupted as a result of viral integration, L1-negative samples were also subject to PCR using E1 primers. To determine specific HPV isoforms, primers to the E7 genes of HPV16, 18, 33 and 52 were used [20–23]. Specificity was determined on template DNA from SiHa (HPV18) and HeLa (HPV16) cell lines, and plasmids containing HPV33 and HPV52 (gifts from Dr. G. Orth and Dr. W. Lancaster, International HPV Reference Centre, Karolinska Institutet, Stockholm).

PCR was carried out with template DNA (100 ng), forward and reverse primers (0.4 µM), MyTaq DNA Polymerase (0.5 µl) in 50 µl. Denatured DNA (94 °C, 3 min) was subject to 35 cycles of PCR (94 °C for 15 s, primer annealing between 50 and 58 °C for 15 s, 72 °C for 15 s), with a final elongation step of 72 °C for 5 min.

### Confirmation of HPV Genotypes by DNA Sequencing

PCR products were purified using the QIAquick Gel Extraction Kit. Briefly, DNA fragments were excised from the agarose gel, solubilised with Buffer QG (50 °C, 10 min) before isopropanol precipitation. DNA was collected on a QIAquick spin column before eluting with dH_2_0. PCR products were sequenced by the Tayside Centre for Genomic Analysis, Ninewells Hospital. PCR products (5 µl) were purified using a modification of the ExoSAP method by incubating with exonuclease I (1 U) and shrimp alkaline phosphatase (1 U) for 20 min at 37 °C before inactivation at 80 °C for 15 min. Samples were sequenced bidirectionally using the ABI BigDye Terminator version 3.1 cycle sequencing kit (Applied Biosystems) and analysed on the Applied Biosystems 3730 DNA Analyser (Applied Biosystems). Sequence analysis was performed using MacVector version 12.03 (MacVector Inc., Waterbeach, Cambridge, UK).

### Statistical Analysis

Data was analysed using SPSS (IBM Statistics Version 22). Data related to categorical variables was described in terms of number of patients (percentages) and as mean or median for continuous variables. Chi-squared test of independence was used to report on pairwise correlation between p16 status, HPV status and EBP50 expression on the one hand and social demographics and clinical/histopathological characteristics of the cohort on the other. Statistical significance was defined as *p* < 0.05. Overall survival (OS) was defined as time (months) from diagnosis to death, end of study or date of last follow-up. Recurrence free survival (RFS) was defined as time (months) from diagnosis to locoregional recurrence. Disease free survival (DFS) was defined as time (months) from diagnosis to death due to head and neck cancer. Kaplan–Meier analysis was used to obtain 2- and 5-year survival curves. The Cox Proportional hazards model was used to estimate Hazard Ratio (HR) and 95% Confidence Interval (CI) for prognostic significance of single and multiple variables. The Likelihood Ratio test was used to compare the relative strength of p16 and EBP50 expression as indicators of HPV infection.

## Results

**Fig. 1:**
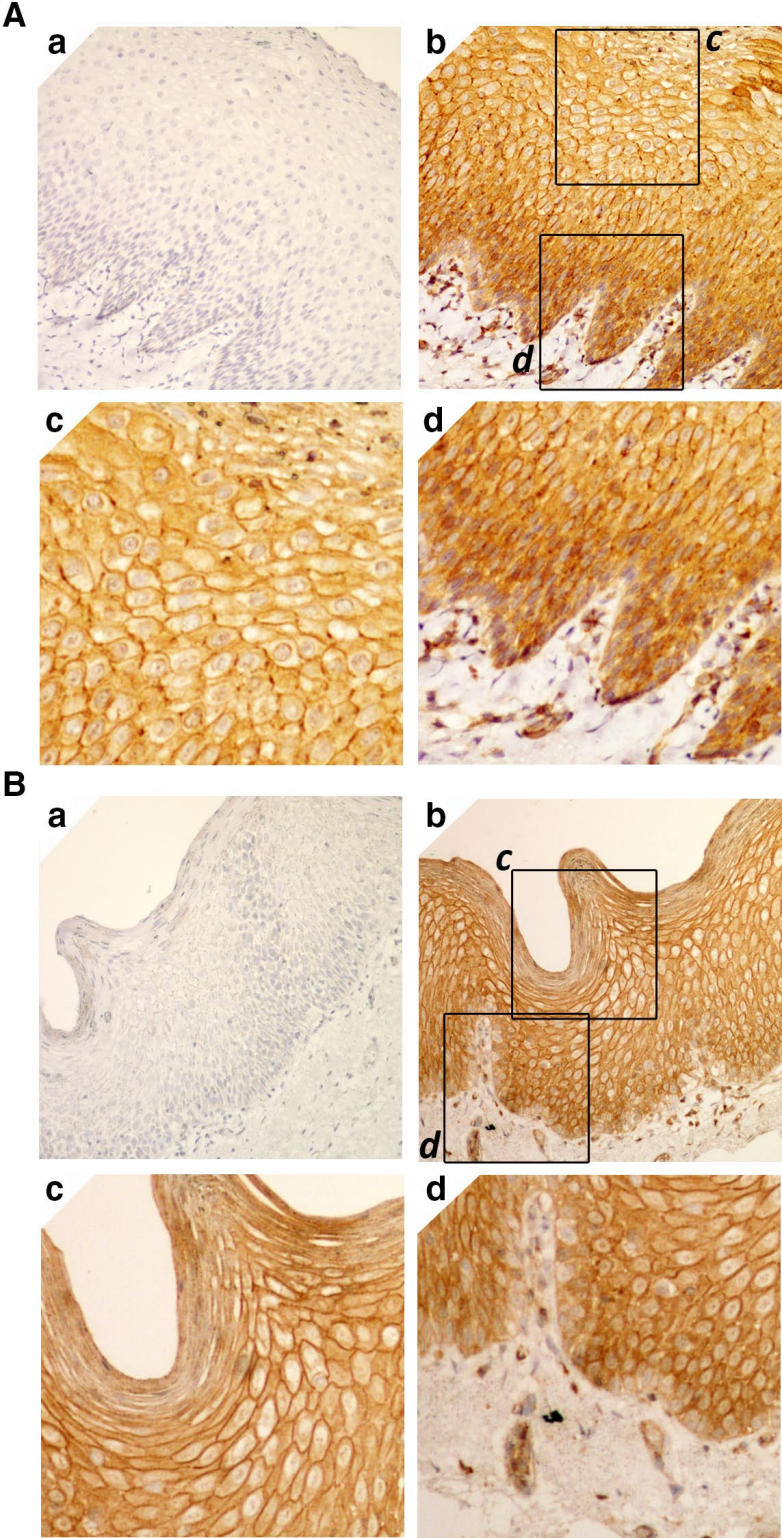
Differential localisation of EBP50 in stratified squamous epithelium of **A** tonsil tissue and **B** normal oral mucosa. Immunohistochemical analysis of oral tissue sections stained with *a* No primary antibody and *b* anti-EBP50 antibody (1:2000), and counterstained with haematoxylin. *c* and *d* are expanded sections of (*b*) showing suprabasal (stratum spinosum, stratum granulosum) and basal layers (stratum basale), with membrane-bound and cytoplasmic EBP50 respectively. Little or no EBP50 staining was visible in the outer stratum corneum. Magnification ×100

Given the heterogeneous nature of the cohort, we undertook immunohistochemistry of FFPE stratified squamous epithelium of tonsil tissue and normal oral mucosa to establish the baseline pattern of EBP50 staining in the various normal oral epithelia. Although EBP50 staining differed in the different layers of tonsil tissue (Fig. 1A (b)) and normal oral mucosa (Fig. 1B (b)), the staining pattern was consistent between the two tissue types. Whilst EBP50 was predominantly membranous in the suprabasal layers (Fig. 1A, B (c)) (stratum spinosum, stratum granulosum), no immunoreactivity was detected in the stratum corneum. Moving deeper from the suprabasal to the stratum basale, EBP50 staining changed from the membrane to a predominantly cytoplasmic location (Fig. 1A, B (d)), which correlated with proliferative basal layers. No nuclear EBP50 immunoreactivity was detected, and antibody specificity confirmed by blocking with recombinant GST-EBP50 protein (data not shown).

Having established the baseline EBP50 staining in oral tissue, a cohort of 156 samples from predominantly male patients (68%) with a mean age of 65 years (range 30–90) were selected. The mean length of follow up i.e. the date of diagnosis to the date of last follow up or the end of the study period of the date of death was 39 months, with 75% of patients followed up for 60 months. Treatment was surgery (19%), radiotherapy (25%), surgery and post-operative radiotherapy (11%), multimodality treatment (35%), palliation (8%) and data missing (2%). The tumour sites were oropharyngeal (37.8%), laryngeal (24%), oral (19%) and other sites (unknown primaries, nares, nasopharyngeal) (18.5%). Approximately half the patients presented with stage IV disease (48%). Recurrence was observed in 16% of cases and the OS was 50.6%.

**Fig. 2:**
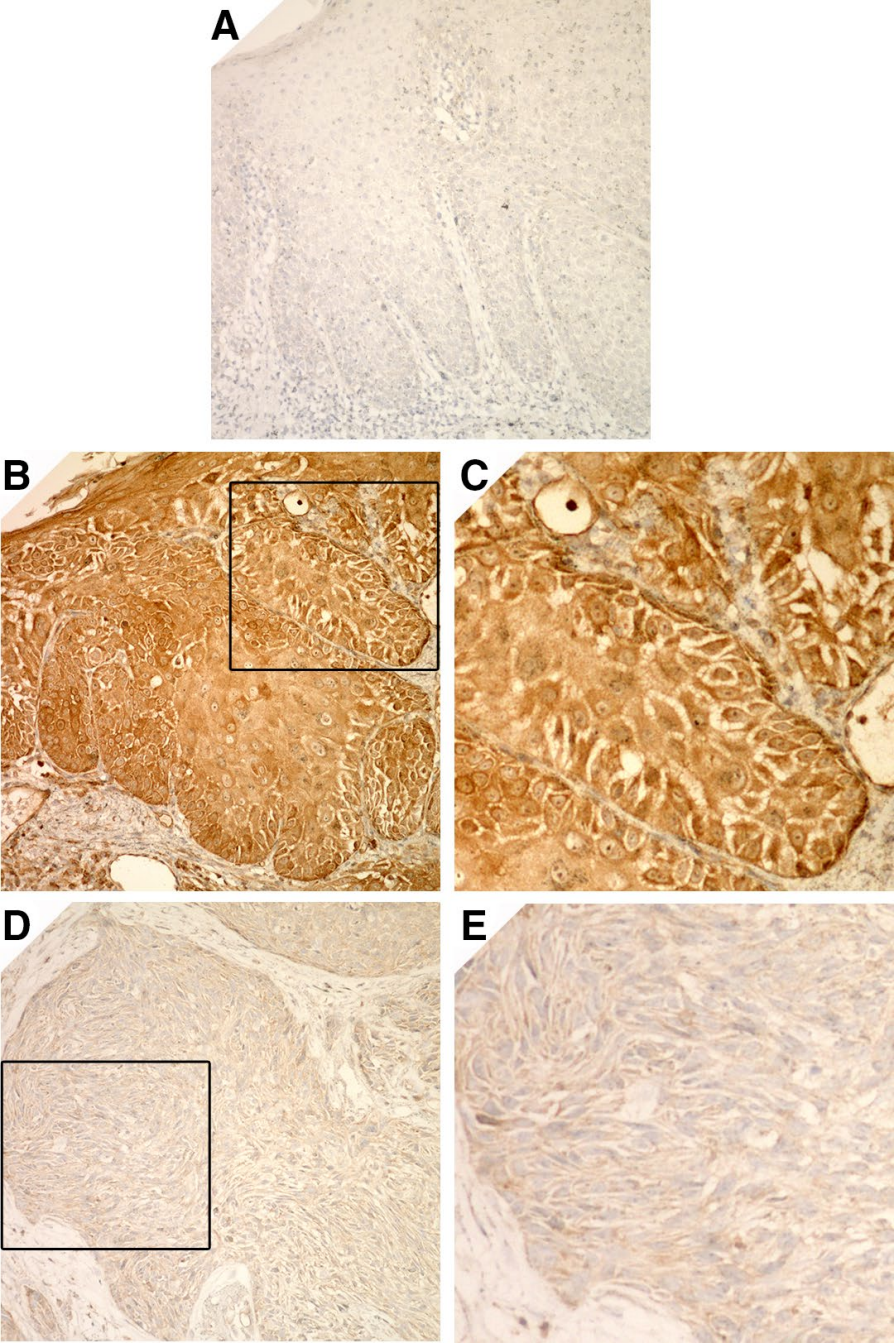
Cytoplasmic EBP50 is primarily found in tumour tissue. Immunohistochemical examples of sections with (**a**) no primary antibody control, counterstained with haematoxylin, or (**b**) tumours having ‘predominantly cytoplasmic’ or (**d**) ‘weak-negligible’ EBP50 staining. ×100 magnification. Panels (**c**) and (**e**) are expanded sections from (**b**) and (**d**), showing cytoplasmic staining of EBP50, with no detectable membrane staining. Magnification ×400

EBP50 expression within the tumour cohort, quantified as described in Table 1, is summarised in Table 2, with representative immunohistochemical examples of patterns found in the majority of HNSCC shown in Fig. 2. The majority of tumours had cytoplasmic EBP50, the breakdown of which was ‘predominantly cytoplasmic’ in 76 (49%) samples (Fig. 2b, c), ‘weak/negligible cytoplasmic’ in 44 (28%) samples (Fig. 2d, e) and ‘strongly cytoplasmic’ in 5 (3%) of samples. ‘Heterogeneous’ staining, where cytoplasmic EBP50 varied across a tumour (negative to positive), occasionally with some sparse membrane staining, was seen in 26 (17%) samples. Five samples (3%) had traces of membranous staining within a cytoplasmic EBP50 background. In general, with the exception of a few samples, HNSCC specimens were immunonegative for membranous EBP50 (Table 2). The lack of EBP50 expression at the plasma membrane of tumour cells (Fig. 2b, c) is consistent with the EBP50 staining in the stratum basale in normal tissue (Fig. 1). Together, these data are consistent with cytoplasmic or low levels of EBP50 being specifically associated with proliferative tissues, both normal and tumour in origin.

**Table 2:** Summary of the immunohistochemical EBP50 staining patterns found in HNSCC in a large Scottish cohort

EBP50 staining	Membrane staining	Cytoplasmic staining	No. of tumours	%
Negligible/weak	–	−/+	44	28
Moderate	–	++	76	49
Strong	–	+++	5	3
Heterogeneous	Traces^a^	Variable^b^	13	17
Heterogeneous	–	Variable^b^	13	
Other	Traces^a^	−/+, ++	5	3

The majority of tumours had cytoplasmic EBP50. In general, with the exception of a few minor examples, HNSCC samples were immunonegative for membranous EBP50

^a^In a few cells within the tumour

^b^Variation in EBP50 staining across a tumour (−/+, ++ or +++)

The clinical and pathological characteristics of the study subset were analysed based on EBP50 expression patterns (Table 3), where we found a significant association with smoking status and disease. ‘Weak/negligible cytoplasmic’ EBP50 expression significantly correlated with non- or ex-smokers (p = 0.019), late stage disease (p = 0.02) originating in the oropharynx (p < 0.001), positive neck nodes, overexpression of p16 and positive HR-HPV DNA status (p < 0.001). In contrast, tumours with predominantly ‘cytoplasmic’ EBP50 expression correlated with patients with a current smoking history, were localised in the oral cavity and larynx, with no p16 overexpression and no positive nodes (p < 0.001).

**Table 3:** Social demographics and clinicopathological characteristics of patients based on EBP50 expression

Variable	EBP50 expression patterns					
	Predominantly cytoplasmic	Weak/negligible cytoplasmic	Hetero-geneous	Other	Total	Pearson’s χ^2^
History of smoking						
Non-smoker	9 (15%)	12 (32%)	5 (22%)	1 (11%)	27	*P* = 0.019
Smoker	36 (60%)	8 (22%)	11 (48%)	6 (67%)	61	
Ex-smoker	15 (25%)	17 (46%)	7 (30%)	2 (22%)	41	
Total	60	37	23	9	129^a^	
History of alcohol consumption						
Light–moderate drinker	33 (43%)	27 (61%)	12 (46%)	3 (30%)	75	*P* = 0.14 (NS)
Heavy drinker	15 (20%)	7 (16%)	8 (31%)	5 (50%)	35	
Non-drinker	8 (10%)	2 (5%)	3 (12%)	0 (0%)	13	
Ex-heavy drinker	2 (3%)	0 (0%)	0 (0%)	1 (10%)	3	
Alcohol status unknown	18 (24%)	8 (18%)	3 (11%)	1 (10%)	30	
Total	76	44	26	10	156	
Site of primary tumour						
Oral cavity	17 (22.4%)	5 (11%)	7 (27%)	1 (10%)	30	*P* < 0.001
Oropharynx	17 (22.4%)	31 (71%)	9 (35%)	2 (20%)	59	
Larynx	23 (30%)	2 (4%)	7 (27%)	6 (60%)	38	
Other	19 (25%)	6 (14%)	3 (11%)	1 (10%)	29	
Total	76	44	26	10	156	
Grade of differentiation						
Well differentiated	1 (1%)	0 (0%)	0 (0%)	0 (0%)	1	*P* = 0.243 (NS)
Moderately differentiated	32 (44%)	7 (18%)	11 (44%)	2 (25%)	52	
Poorly differentiated	33 (45%)	26 (67%)	12 (48%)	6 (75%)	77	
Moderate–poorly differentiated	7 (10%)	6 (15%)	2 (8%)	0 (0%)	15	
Total	73	39	25	8	145^a^	
Disease stage						
Stage I	13 (18%)	3 (7%)	4 (18%)	3 (30%)	23	*P* = 0.02
Stage II	12 (17%)	1 (2%)	3 (13%)	2 (20%)	18	
Stage III	12 (17%)	4 (9%)	6 (26)	3 (30%)	25	
Stage IVA & IVB	33 (45%)	32 (73%)	9 (39%)	1 (10%)	75	
Unknown	2 (3%)	4 (9%)	1 (4%)	1 (10%)	8	
Total	72	44	23	10	149^a^	
Neck node status						
Negative	43 (59%)	5 (11%)	14 (54%)	8 (80%)	70	*P* < 0.001
Positive	30 (41%)	39 (89%)	12 (46%)	2 (20%)	83	
Total	73	44	26	10	153^a^	
p16 status						
Positive	7 (9%)	32 (73%)	3 (11%)	2 (20%)	44	*P* < 0.001
Negative	69 (91%)	12 (27%)	23 (89%)	8 (80%)	112	
Total	76	44	26	10	156	
HPV status						
Positive	6 (9%)	29 (83%)	3 (12%)	1 (11%)	39^c^	*P* < 0.001
Negative	63 (91%)	6 (17%)	22 (88%)	8 (89%)	99	
Total	69	35	25	9	138^b^	
OS						
Alive	31 (41%)	27 (61%)	15 (58%)	6 (60%)	79	*P* = 0.12 (NS)
Dead	45 (59%)	17 (39%)	11 (42%)	4 (40%)	77	
Total	76	44	26	10	156	
Recurrence						
Yes	18 (24%)	5 (11%)	1 (4%)	1 (10%)	25	*P* = 0.067 (NS)
No	58 (76%)	39 (89%)	25 (96%)	9 (90%)	131	
Total	76	44	26	10	156	

Significant associations were noted between EBP50 expression and smoking history, site of primary tumour, stage of disease, metastasis to cervical lymph nodes, tumour p16 and HPV status

^a^Patients with missing or unavailable data were excluded from analysis

^b^Patients with ‘Equivocal’ HPV status were excluded from analysis

^c^EBP50 staining could not be determined in one HPV-positive specimen

No significant associations were found between EBP50 staining patterns and grade of tumour differentiation, alcohol consumption, OS or recurrence. Patients with ‘weak/negligible cytoplasmic’ EBP50 expression had the highest OS and RFS compared to those with ‘predominantly cytoplasmic’ EBP50 expression (OS—Median 49 months, 50 vs. 68%; 5 years, 46 vs. 60%, *P* > 0.05; RFS—5 years, 64 vs. 87%, *P* = 0.04) (data not shown). When the impact of clinical and histological variables on RFS was investigated through univariate survival analysis, smoking status at diagnosis (*P* = 0.001), and EBP50 expression (*P* = 0.04) were found to be significantly associated with RFS.

In total, 40 samples were positive for HR-HPV DNA (25%) and 44 were p16 positive (28%). Of the HPV-positive samples, 39 were HPV16 positive and one was HPV18 positive. From the anatomic breakdown, the majority of HPV-positive tumours (83%) arose in oropharyngeal sites, whilst 17% arose in the oral cavity/other sites (Table 4). For the purposes of this study, a specimen was HPV positive if it was both p16 and HPV DNA positive. To compare p16 and EBP50 expression as indicators of HPV infection, the relative strength of their dependence with HPV DNA status was tested. Two independent Pearson’s Chi Squared tests (χ^2^) were performed to test the dependence between EBP50 expression, HPV DNA status and p16 expression and HPV DNA status (Table 5). Each test showed a strong correlation between HPV DNA status and the indicator variables. Since the *P* values for both tests were identical (*P* < 0.001), the strength of goodness of fit of the variables was compared. The Likelihood Ratio for p16 status (87) was marginally higher than EBP50 expression (79.4).

**Table 4:** Summary of HPV-positive tumours by anatomic location in the head and neck

Head and neck subsite group	Clinical anatomic location	No. of patients	Type of high-risk HPV
Oral cavity	Dorsum of tongue	1	HPV 16
	Palate	1	HPV 16
	Anterior 1/3 of tongue	1	HPV 16
Oropharynx	Base of tongue	9	HPV 16
	Tonsil	24	HPV 16
Other	Unknown primary	1	HPV 16
	Pharynx	1	HPV 16
	Nares	1	HPV 16
	Nasal cavity	1	HPV 18

The majority of HPV-positive tumours (33/40; 83%) arose from the oropharynx with the tonsils and base of the tongue being the most common subsites of origin. An overwhelming majority of the HPV-positive tumours were infected with high-risk type 16

**Table 5:** EBP50 expression and p16 status correlation with HPV DNA status using Pearson’s χ^2^ tests

HPV DNA status (n = 156)	p16 Overexpression		*P* value and likelihood ratio value	EBP50 expression		*P* value and likelihood ratio value
	Positive	Negative		Weak/negligible	Other	
Positive	39 (75%)	13 (25%)	*P* < 0.001 87	38 (73%)	14 (9%)	*P* < 0.001 79.4
Negative	5 (5%)	99 (95%)		6 (6%)	98 (63%)	

Two independent Pearson’s χ^2^ tests showed strong correlation between HPV DNA status and both p16 overexpression and EBP50 expression pattern

## Discussion

A number of studies have reported on the potential role of EBP50 in carcinogenesis, including breast [24, 25], liver [6], colorectal [5], and gastric cancers [19]. Here, we report for the first time a study on EBP50 expression, and the impact of HR-HPV on EBP50 expression in HNSCC tumours in order to establish its validity as a novel biomarker of HR-HPV infection in HNSCC.

**Fig. 3:**
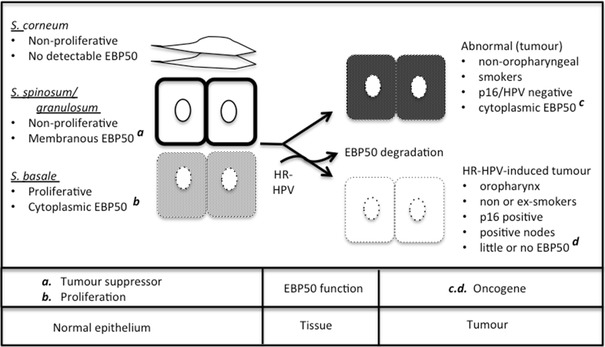
Subcellular localisation and levels of EBP50 define a distinct patient profile in HNSCC and may determine its function. In normal epithelium, EBP50 is present on the membrane (thick black line) in suprabasal layers (S. spinosum, S. granulosum) (*a* tumour suppressor function), whilst in the basal layers (light shading), it is present in the cytoplasm (*b* proliferative function). In diseased tissue, high levels of cytoplasmic EBP50 (darker shading) are predominantly found in non-oropharyngeal HNSCC, whilst HR-HPV associated tumours, which are predominantly found in the oropharynx, have little or no EBP50

Collectively, our data support a model whereby the level and subcellular distribution of EBP50 may determine its function, define distinct patient profiles and identify loss of EBP50 as a potential surrogate biomarker for HR-HPV infection in oropharyngeal tumours (Fig. 3). Cytoplasmic EBP50 was found in the stratum basale in normal tissue (Fig. 3b) and in the majority of tumours (Fig. 3c), consistent with a role in this predominantly proliferative pool of epithelial cells [4, 25]. In contrast, membrane-associated EBP50 was found in the suprabasal layers (Fig. 3a), but absent from the basal layer (Fig. 3b) and tumour sections (Fig. 3c), consistent with a role as a tumour suppressor. Contrary to others, we found no conclusive evidence for nuclear EBP50, which has been reported to regulate the Wnt/β-catenin pathway [26, 27], and be a prognostic marker in breast cancer [28].

Two distinct patient profiles in this cohort were revealed when the relationship between EBP50 expression patterns and clinicopathologic characteristics was analysed, with significant associations noted with smoking history at diagnosis, site of primary tumour, disease stage, lymph node status, p16 status and HPV status (Fig. 3). The majority of tumours with ‘predominantly cytoplasmic’ EBP50 were more likely to be patients with a smoking history, with p16/HR-HPV-negative tumours arising in non-oropharyngeal sites such as the oral cavity and larynx with no involvement of neck nodes (Fig. 3c). In contrast, patients who were either non-smokers or ex-smokers at the time of diagnosis, had p16/HR-HPV-positive oropharyngeal tumours, presented with positive neck nodes, late stage disease, and were more likely to have ‘weak/negligible cytoplasmic’ EBP50 expression (Fig. 3d), suggesting these may have a different pathology from those expressing ‘predominantly cytoplasmic’ EBP50. The improved survival in this cohort is in keeping with other published work in this area suggesting that although these patients have aggressive disease they respond well to treatment [13]. HR-HPV E6 oncoprotein has the potential to interact with and mediate the degradation of a subset of PDZ-containing cell polarity regulators including human Dlg [29, 30] and EBP50 [9], and specific subcellular pools of cell polarity proteins [31, 32]. Consequently, reduced EBP50 expression in the group of patients with oropharyngeal tumours may be a result of HR-HPV E6-mediated degradation of membrane pools of EBP50 (Fig. 3d), thereby disrupting cell polarity which is central to the control of cell proliferation and cell survival [10].

The relationship between EBP50 expression as an independent variable and clinical outcomes of patients in the cohort showed that patients with ‘predominantly cytoplasmic’ EBP50 expression had the worst prognosis whilst reduced cytoplasmic EBP50 expression was associated with better OS and RFS. Previous reports on clinical outcomes and EBP50 expression are varied, seemingly linked to subcellular localisation of the protein [3]. In invasive breast carcinomas, cytoplasmic EBP50, high or low, did not correlate with OS or DFS [28]. Likewise, in gastric cancer no correlation was found between OS rates and EBP50 expression [19]. In our cohort, a marked improvement was observed in the DFS survival rates for the patients with ‘weak/negligible cytoplasmic’ EBP50-expressing tumours, while the survival rate of patients with ‘predominantly cytoplasmic’ EBP50 steadily declined over time. Since DFS analysis was not statistically significant, EBP50 expression may not be a valuable indicator of prognosis in patients with HNSCC.

The molecular mechanisms, which are known to regulate the subcellular localisation of EBP50 and as such its function, are varied, ranging from phosphorylation or interaction with ERM proteins [3]. Functionally, membrane-associated EBP50 has been shown to regulate the adherens junction proteins E-cadherin and β-catenin at the cell surface [8]. In addition, EBP50 regulates PTEN, a negative regulator of PI-3K/AKT signalling at both the membrane and in the cytoplasm [3, 7]. Although we cannot attribute a function with the redistribution of EBP50 to the cytoplasm in our cohort, collectively, our data are consistent with a tumour suppressor function at the cell membrane, and oncogenic function in the cytoplasm (Fig. 3), possibly by regulating the PI-3K/AKT/PTEN axis in the cytoplasm and Wnt/β-catenin signalling through the adherens junction proteins E-cadherin and β-catenin at the cell membrane [3, 7, 8].

## Conclusion

In the majority of oral cancers, EBP50 expression was predominantly cytoplasmic. A smaller group of tumours, the majority involving the oropharynx, demonstrated reduced/negligible cytoplasmic EBP50 expression, which strongly correlated with a positive HR-HPV status. These data suggest that EBP50 may act as a tumour suppressor when localised at the plasma membrane or act as an oncogene when relocalised to the cytoplasm or its expression is lost. In addition, since EBP50 expression differs between oropharyngeal and non-oropharyngeal tumours, this may be linked to its degradation by HR-HPV and as such, could serve as a surrogate marker for HR-HPV.
